# Cryo-EM structure of the glycosylated protein CgeA in the crust of *Bacillus subtilis* endospores

**DOI:** 10.71150/jm.2504013

**Published:** 2025-10-31

**Authors:** Migak Park, Doyeon Kim, Yeongjin Baek, Eunbyul Jo, Jaekyung Hyun, Nam-Chul Ha

**Affiliations:** 1 Research Institute of Agriculture and Life Sciences, Department of Agricultural Biotechnology, CALS, Seoul National University, Seoul 08826, Republic of Korea; 2 Center for Food and Bioconvergence, Department of Agricultural Biotechnology, Interdisciplinary Programs in Agricultural Genomics, CALS, Seoul National University, Seoul 08826, Republic of Korea; 3 School of Pharmacy, Sungkyunkwan University, Suwon 16419, Republic of Korea

**Keywords:** *Bacillus subtilis*, spore-forming bacteria, cryo-electron microscopy, crust protein, CgeA

## Abstract

The *Bacillus subtilis* spore crust is an exceptionally robust proteinaceous layer that protects spores under extreme environmental conditions. Among its key components, CgeA, a glycosylation-associated protein, plays a critical role in modifying crust properties through its glycosylated moiety, enhancing spore dispersal in aqueous environments. In this study, we present the high-resolution cryo-electron microscopy structure of the core region of CgeA at 3.05 Å resolution, revealing a doughnut-like hexameric assembly. The N-terminal regions are disordered, whereas the C-terminal region forms the core of the hexamer. Although the loop containing Thr112 was not resolved in the density map, its location can be inferred from surrounding residues, suggesting that Thr112 is situated on the exposed surface of the hexamer. On the opposite face, a distinct electrostatic pattern is observed, featuring a negatively charged central pore and a positively charged outer surface. Modeling and biochemical studies with the putative glycosyltransferase CgeB provide insights into how the glycosyl group is transferred to Thr112. This study offers a molecular-level understanding of the assembly, glycosylation, and environmental adaptability of the *B. subtilis* spore crust, with valuable implications for controlling spore formation in industrial applications.

## Introduction

*Bacillus subtilis* forms highly resilient spores as a survival strategy under extreme environmental stress or nutrient deprivation (Cohn, 1875). These spores, capable of dispersing across diverse environments, facilitate the colonization of new hosts and ensure the long-term survival of the bacteria (Nicholson et al., 2000). In addition to natural ecosystems, *B. subtilis* spores are frequently detected in food production and processing environments. Their exceptional resistance allows them to persist in dried food products, seasonings, powdered milk, and other food matrices, often surviving conventional food preservation techniques such as heat treatment and desiccation (Amador Espejo et al., 2014; Banks et al., 1988). Spore formation is also a major concern in foodborne pathogens, such as *B. cereus* and *B. anthracis*, both associated with foodborne illnesses (Kramer and Gilbert, 1989). Therefore, understanding the structural characteristics and formation mechanisms of spores is critical for developing effective microbial control strategies in the food industry.

Sporulation is a complex multistage process. In response to environmental cues, cells undergo asymmetric division, producing a smaller prespore (forespore) and a larger mother cell. The mother cell then engulfs the prespore in a phagocytosis-like manner, fully enclosing the spore (Earl et al., 2008). As sporulation progresses, multiple protective layers form around the prespore, including the spore crust, which is the outermost proteinaceous barrier that shields spore from environmental extremes (Henriques and Moran, 2007; McKenney et al., 2013).

The spore crust, along with other structural layers such as the cortex and coat (Atrih and Foster, 1999), plays a vital role in protecting spores against heat, desiccation, and mechanical stress (McKenney et al., 2010; Zhang et al., 1993). It primarily comprises proteins, including CotV, CotW, CotX, CotY, CotZ (Zhang et al., 1993), and CgeA and CgeB (Roels and Losick, 1995). Operon structure analysis has revealed that *cotVWXYZ* genes are clustered together, whereas *cgeA* is encoded on a separate chromosome. CotY—a cysteine-rich protein—forms hexagonal sheets that provide structural support for the crust (Jiang et al., 2015). CotZ anchors to CotY to ensure proper localization across the spore surface (Imamura et al., 2011).

Unlike CotY and CotZ, CgeA is less abundant and does not contribute to the structural integrity of the crust. Instead, CgeA plays a distinct role in modifying spore surfaces through glycosylation. This modification is thought to be facilitated by putative glycosyltransferases such as CgeB and CgeD, which are encoded in the *cgeA*B and *cgeCDE* operons, respectively (Roels and Losick, 1995; Shuster et al., 2019b). CgeA has been suggested to be glycosylated at Thr112 (Nakaya et al., 2023), although the complete glycosylation pattern remains unclear. This glycosylation process reduces the hydrophobicity of the spore surface and enhances spore dispersal in aqueous environments by altering crust properties.

Spore dispersal is a key factor in microbial ecology and has significant implications for food production. *Bacillus* spores can adhere to food-processing equipment and production lines, and resuscitate. Once dispersed and deposited on surfaces, spores may germinate and contribute to the formation of biofilms, which can withstand standard cleaning and sanitation procedures (Lindsay et al., 2002, 2006). In food processing environments, this change in hydrophobicity due to glycosylation may affect spore adhesion to equipment surfaces and influence biofilm formation. Investigating the molecular mechanisms underlying CgeA glycosylation may provide valuable insights into spore persistence and spread in food-related settings.

Sequence analysis suggests that CgeB is a putative glycosyltransferase responsible for modifying CgeA (Bartels et al., 2019). Shuster et al. (2019a) have reported that spores of Δ*cgeA* and Δ*cgeB* mutant strains exhibit increased surface hydrophobicity compared with those of the wild-type strain. India ink staining has revealed that the polysaccharide layer is significantly reduced or entirely absent in these mutants. Transmission electron microscopy (TEM) has further demonstrated that the spore crust in these mutants is either loosely attached or missing altogether (Shuster et al., 2019a).

Despite the functional importance of CgeA, the precise mechanisms through which it participates in glycosylation and crust assembly are incompletely understood. In this study, we present the cryo-electron microscopy (cryo-EM) structure of the core region CgeA at 3.05 Å resolution. The structure reveals a homohexameric assembly with a doughnut-like shape. The flexible loop spanning residues 110–114 was not resolved in the density map, but the surrounding structured residues up to positions 109 and 115 suggest that the glycosylation target residue Thr112 (Nakaya et al., 2023) is positioned on the exposed surface of the hexamer. A complex model involving the putative glycosyltransferase, CgeB, suggests that glycosylation is specifically transferred to CgeA. These findings provide a structural framework for understanding the molecular basis of the hydrophilic decoration of endospore surfaces, which facilitates spore dispersal in aqueous environments.

## Materials and Methods

### Plasmid construction

The *cgeA* gene from *B. subtilis* was synthesized by Gene Synthesis Services (Bionics, Korea). Using primers designed to incorporate NcoI and XhoI restriction sites, the synthesized gene was subcloned into the corresponding sites of the pProEx-Hta expression vector (Invitrogen, USA), generating pProEx-Hta-CgeA (WT). Primer sequences used for polymerase chain reaction (PCR) are listed in Table S1. To generate the CgeA A42C/D97C variant, point mutations were introduced using the PCR-based QuikChange protocol (Agilent Technologies, USA) with primers specified in Table S1. The recombinant pProEx-HTa vector carrying these mutations was designated pProEx-Hta-CgeA (A42C/D97C). These vectors contained an N-terminal 6×His tag for protein purification.

### Protein expression and purification

The pProEx-Hta-CgeA (WT) and pProEx-Hta-CgeA (A42C/D97C) plasmids were transformed into *Escherichia coli* BL21 (DE3) competent cells. Transformed cultures were grown in Luria bertani (LB) broth (1.5 L) containing 100 μg/ml ampicillin at 37°C until the optical density at 600 nm reached 0.6. Protein expression was induced by adding 0.5 mM isopropyl-β-D-thiogalactoside to the culture medium, followed by incubation at 30°C for 6 h. Bacterial cells were harvested by centrifugation at 5,500 × g. The harvested *E. coli* expressing CgeA were resuspended in 50 ml lysis buffer containing 20 mM Tris-HCl (pH 8.0) and 150 mM NaCl. Cells were lysed using a French press (Constant Systems Ltd., UK) operating at 23 kpsi. Cell debris was removed by centrifugation at 20,000 × g for 30 min at 4°C. The cell lysate was incubated with Ni-NTA agarose resin (Qiagen, Germany) with gentle rolling in a column (GE Healthcare, UK). The resin was washed with lysis buffer supplemented with 20 mM imidazole, and bound protein was eluted using 40 ml buffer containing 250 mM imidazole. To remove the 6×His tags, eluted proteins were treated with recombinant Tobacco Etch Virus protease. The protein solution was then diluted three-fold with 20 mM Tris-HCl (pH 8.0) buffer and subjected to anion-exchange chromatography using a HiTrap Q column (GE Healthcare) with a 0–1 M NaCl gradient. The eluted protein was further purified using size-exclusion chromatography on a HiLoad 16/600 Superdex 200 pg column (Cytiva) pre-equilibrated with 20 mM Tris-HCl (pH 8.0) and 150 mM NaCl. The final purified protein was concentrated to 10 mg/ml and stored at −80°C until use.

### Size-exclusion chromatography coupled with multiangle light scattering (SEC-MALS)

Wild-type CgeA protein in a buffer containing 20 mM Tris-HCl (pH 8.0) and 150 mM NaCl was used for SEC-MALS analysis. Each sample was subjected to SEC using a Superdex 200 Increase 10/300 GL column (GE Healthcare). The molecular size and oligomerization state of CgeA were measured using MALS (DAWN HELIOS II; Wyatt Technology, USA). The data were analyzed using ASTRA 6 software (Wyatt Technology).

### Negative-stain EM of CgeA protein

Ten microliter aliquot of 10 µM CgeA protein was applied to freshly glow-discharged, 400-mesh carbon-coated copper grids (Electron Microscopy Sciences, USA). The adsorbed protein was subjected to negative staining with a 1% (w/v) uranyl acetate solution and air dried at 25°C. Negative-stain EM imaging was performed using a 120 kV Tecnai G2 Spiri TWIN microscope (FEI, USA) equipped with a Rio 4 CMOS camera (Gatan Inc., USA). The analysis was performed at the Center for Macromolecular and Cell Imaging, Seoul National University.

### Cryo-EM data collection

For Wild-type CgeA, 3 µl aliquot of 0.2 mg/ml CgeA protein was applied to Quantifoil R 1.2/1.3 400 mesh copper grids (Electron Microscopy Sciences). These grids were blotted for 5 s with a blot force of 0 at 4°C and subsequently plunge-frozen in liquid ethane using a Vitrobot Mark IV system (Thermo Fisher Scientific Inc., USA). Cryo-EM micrographs were acquired using a 200 kV Glacios cryo-TEM microscope (Thermo Fisher Scientific) equipped with an extreme-field emission gun (X-FEG), Ceta 16M camera, and Falcon 4 camera. A total of 1,952 movies were collected at a nominal magnification of ×92,000 (resulting in a pixel size of 1.1 Å) and a total dose rate of 49.97 e Å^−2^ using EPU automatic data acquisition software (Thermo Fisher Scientific). An exposure time of 14.62 s was employed. The resulting videos were saved in MRC format. A defocus range of −2.0 to −1.0 μm was utilized (Table S2).

For the CgeA A42C/D97C mutant, 3 µl aliquot of CgeA A42C/D97C protein (0.1 mg/ml, 0.2 mg/ml) was applied to Quantifoil 1.2/1.3 300 mesh copper grids. These grids were blotted for 5 s with a blot force of 0 at 4°C and subsequently plunge-frozen in liquid ethane using a Vitrobot Mark IV system. Cryo-EM micrographs were acquired using a 300 kV Krios G4 cryo-TEM microscope (Thermo Fisher Scientific) equipped with an X-FEG, a Ceta CMOS camera, and a Gatan K3 BioQuantum Detector. A total of 10,594 movies were collected at a nominal magnification of ×105,000 (resulting in a pixel size of 0.828 Å) and a total dose rate of 63.6 e Å^−2^ using automatic data acquisition software. An exposure time of 5.45 s was employed, and the resulting videos were saved in MRC format. A defocus range of −1.7 to −0.8 μm was utilized (Table S2).

### Data processing

For Wild-type CgeA, the cryo-EM dataset was processed using CryoSPARC v4 (Punjani et al., 2017). In total, 1,952 movies were imported into the CryoSPARC server, and the contrast transfer function (CTF) of the prepared micrographs was estimated. Subsequently, approximately 200 ring-like CgeA structures were manually selected, and two-dimensional (2D) classification was performed to extract these images. Auto-picking was conducted using a template picker, which identified 3,559,545 particles. Further refinement involved selecting 2D classified images in which the ring-like structure of CgeA was recognizable, enabling the construction of a three-dimensional (3D) electron density map using cryo-EM. The final map was obtained at a resolution of 6.08 Å, as determined by the Fourier shell correlation (FSC) at 0.143.

For CgeA A42C/D97C mutant, the cryo-EM dataset was processed using CryoSPARC v4.6.0 (Punjani et al., 2017). In total, 10,594 movies were imported into the CryoSPARC server, and the CTF of the prepared micrographs were estimated for defocus correction. Initial particle selection was performed using blob picking to identify approximately 10 nm particles corresponding to the CgeA assemblies. A total of 436,793 particles were automatically selected and subjected to 2D classification to extract ring-like CgeA structures. Subsequently, template picking was performed using the classified 2D images as references. The particles were extracted and subjected to 2D classification to separate top and side views of the structures. Well-defined 2D classes were selected and 3D reconstruction was performed. Heterogeneous and homogeneous refinements were performed with C6 symmetry, followed by local refinement with C6 symmetry. The final map was obtained at a resolution of 3.05 Å, as determined by the FSC at 0.143.

### Model building and refinement

An atomic model of CgeA was constructed using AlphaFold 3 (Abramson et al., 2024) and modified using Coot 0.9.8.93 (Emsley et al., 2010). Refinement was performed using the real-space refinement program in Phenix 1.21 (Adams et al., 2010). Figures were prepared using PyMOL 3.1.3 (Schrödinger, 2015) and ChimeraX 1.9 (Pettersen et al., 2021).

### Glycosyltransferase activity assay

For in vitro glycosylation, CgeA (100 µM) and CgeB (20 µM) were mixed in a reaction buffer containing 20 mM Tris-HCl (pH 8.0), 150 mM NaCl, and 10 mM MgCl_2_. The reaction was initiated by 10 mM UDP-glucose and incubation of the mixture at 37°C for 18 h. Following incubation, the reaction was quenched by adding sodium dodecyl sulfate polyacrylamide gel electrophoresis (SDS-PAGE) loading buffer and 10 μl aliquots of each sample was loaded for SDS-PAGE analysis. 

## Results

### Homohexameric assembly of CgeA protein from *B. subtilis*

**Fig. 1.**
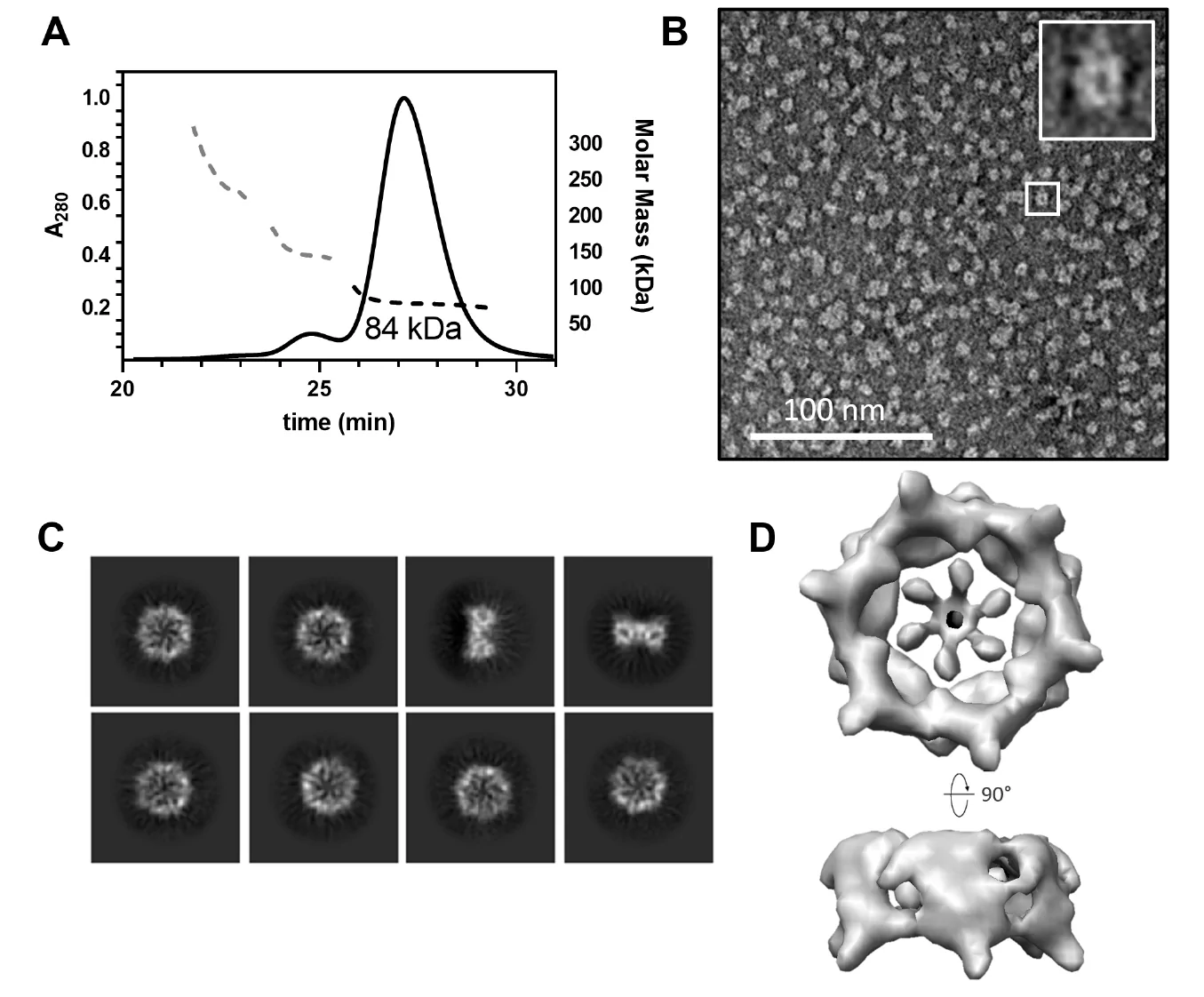
Hexameric assembly of *Bacillus subtilis* wild-type CgeA. (A) Size-exclusion chromatography coupled with multiangle light scattering (SEC-MALS) profiles of the purified CgeA proteins. The ultraviolet absorbance at 280 nm (A_280_ at the left Y-axis) of the SEC is represented by solid lines. The molecular mass (the right Y-axis) based on MALS is represented by a dotted line. The average molar mass (84 kDa) of CgeA is indicated under the dotted lines. (B) Representative negative-stain transmission electron micrographs of the CgeA proteins. The box within the micrographs is enlarged in the top right corner of the micrographs. The scale bar indicates 100 nm. (C) Representative 2D-class averages of CgeA protein from cryo-EM. (D) Electron microscopy map of the CgeA resolved at 6.8 Å. Data collection and processing statistics are presented at Table S2.

The CgeA protein from *B. subtilis* was successfully overexpressed in its soluble form in *E. coli*. Following cell lysis and centrifugation, the protein was purified using a series of chromatographic techniques to obtain a highly soluble preparation of CgeA. Given the limited number of studies on the oligomeric state of CgeA, we analyzed its assembly using SEC-MALS. The analysis revealed that CgeA predominantly assembles as a homohexamer (Fig. 1A). This finding was further corroborated by negative-stain EM, which showed that purified CgeA forms discrete particles (Fig. 1B). The particles were primarily observed in the top-view orientation, clearly displaying a doughnut-like structure and central pores.

To obtain a high-resolution 3D structure of the CgeA protein, we performed cryo-EM analysis of purified protein samples. In total, 1,952 cryo-EM micrographs of vitrified CgeA particles were collected and processed using cryoSPARC v4 (Punjani et al., 2017). Image preprocessing steps, including motion correction, CTF estimation, and particle picking, were performed, followed by 2D classification to isolate high-quality particles (Fig. 1C). These selected particles were then subjected to 3D reconstruction with C6 symmetry imposed, yielding a final 3D map at 6.8 Å resolution (Figs. 1D and S1). The resulting map corresponded to the hexameric core of CgeA, with the N-terminal regions largely absent, likely owing to their intrinsic flexibility. However, we were unable to obtain an atomic model of the CgeA hexamer. This limitation is likely attributable to the insufficient resolution of the cryo-EM map, compounded by the structural flexibility, which hindered the generation of a high-quality electron density map for model building.

### AlphaFold 3-predicted model of CgeA

**Fig. 2.**
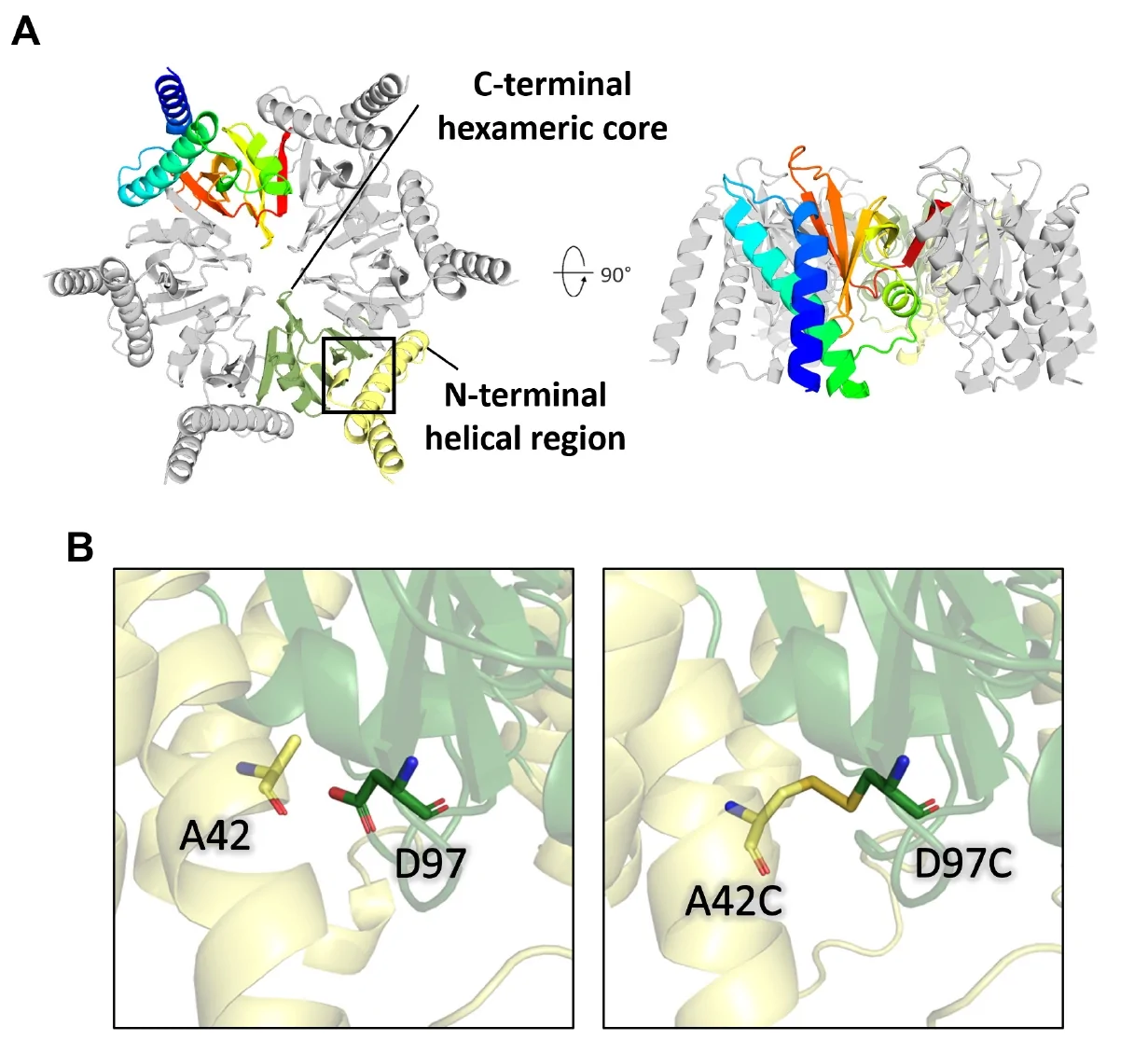
AlphaFold 3-predicted model of CgeA hexamer. (A) Homohexameric model of CgeA predicted by AlphaFold 3 server (Abramson et al., 2024). The N-terminal helical region (residues 1–60) is shown in light yellow, while the C-terminal hexameric core domains (residues 61–133) are shown in green. One subunit within the hexamer is colored in a rainbow gradient from blue (N-terminus) to red (C-terminus) to illustrate the domain organization. The boxed region highlights areas that are shown in greater detail in (B). (B) A close-up view of the box of (A). highlights the predicted proximity between Ala42 and Asp97 (left). AlphaFold 3 predicts a disulfide bond between the mutated cysteine residues in the A42C/D97C mutant of CgeA (right).

AlphaFold 3 (Abramson et al., 2024) was utilized to predict structural models of CgeA using six copies of its amino acid sequence (Fig. 2A). Analyses of the predicted alignment error (PAE) and inter-chain predicted template modeling (ipTM) scores indicated a hexameric configuration, as shown in Fig. S2A. The AlphaFold 3-predicted structure revealed two distinct regions: an N-terminal helical region (residues 1–60) and a C-terminal hexameric core (residues 61–133). The N-terminal helical region contains two elongated alpha-helices and exhibits low predicted local distance difference test (pLDDT) values, suggesting high flexibility and dynamic behavior at the periphery of the hexamer. The C-terminal region harbors a stable domain structure, as evidenced by the high pLDDT values (> 90; Fig. S2A).

These N-terminal helices are connected to the C-terminal rigid core through flexible loops in the AlphaFold 3 model, suggesting a biologically relevant degree of intrinsic flexibility. In the full-length AlphaFold 3-predicted model, the flexible N-terminal helices make molecular contact with the hydrophobic residues in the C-terminal core region. Additionally, these flexible regions are enriched with hydrophilic residues, such as Ser2, Glu4, Asn5, Gln7, Lys9, Asp11, and Thr24, which decorate the hydrophilic surface of the full-length CgeA model (Fig. S2B). Notably, Ala42 in the N-terminal helix and Asp97 in the C-terminal domain of the AlphaFold 3-predicted model were positioned sufficiently close to form a disulfide bond. Therefore, the introduction of cysteine mutations at these positions may facilitate disulfide bond formation under favorable conditions (Fig. 2B).

### Structural determination of CgeA by cryo-EM

To improve the cryo-EM analysis of CgeA proteins, two point mutations (A42C/D97C) were introduced into the CgeA construct to facilitate disulfide bond formation for anchoring the N-terminal helices to the C-terminal core domain based on the AlphaFold 3-predicted model. The engineered CgeA mutant was successfully overexpressed in *E. coli* as a soluble protein and purified using the same protocol as that used for the wild-type protein. Size-exclusion chromatography revealed that the mutant was similar in size to the wild-type protein, confirming that the mutant also assembled into a hexamer (Fig. S3).

**Fig. 3.**
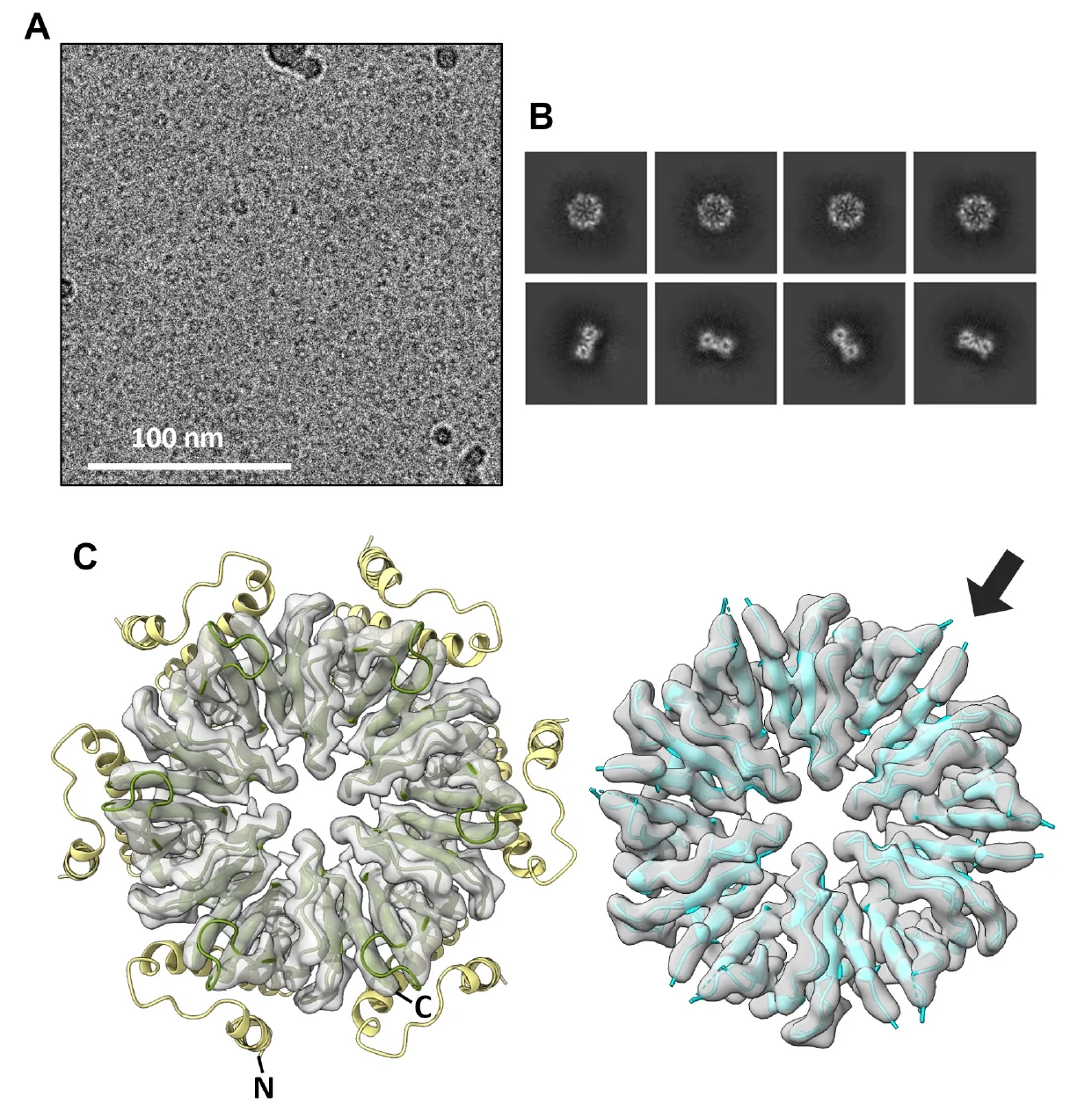
Structural determination of the CgeA A42C/D97C mutant protein by cryo-electron microscopy (cryo-EM). (A) Representative cryo-EM micrograph from the dataset. The scale bar represents 100 nm. (B) Representative 2D-class averages from cryo-EM. (C) Structural comparison of the AlphaFold 3-predicted model (left) and the experimentally solved atomic model (right) of the CgeA hexamer, superimposed onto the electron density map. The N-terminal α-helical region, predicted by AlphaFold 3 but not observed in the map, is shown in light yellow, while the C-terminal core region, visible in the map, is shown in green. The right panel colored in cyan shows only the C-terminal region, which was structurally determined. The arrow indicates a weak density region, which is not visible at the current contour level.

Cryo-EM analysis of the CgeA mutant protein was performed for high-resolution structural reconstruction. A dataset comprising 10,594 movies was processed using cryoSPARC v4.6.0 (Punjani et al., 2017). In our cryo-EM dataset, we observed CgeA mutant particles with central holes and diameters of approximately 10 nm (Fig. 3A). This morphology closely resembles that observed in wild-type CgeA. Motion correction and CTF estimation were followed by particle selection, 2D classification (Fig. 3B), and iterative 3D refinement. The final reconstruction, refined with C6 symmetry, achieved a resolution of 3.05 Å as determined by FSC at the 0.143 threshold (Fig. S4C). However, the model-to-map FSC dropped to 0.5 at 4.0 Å, suggesting a practical model resolution of ~4 Å (Fig. S4D).

The initial atomic model was constructed from the C-terminal domain region of the AlphaFold 3-predicted structure. The model was then iteratively refined using real-space refinement, with manual adjustments performed between refinement cycles. The final refined structure includes residues 62–109 and 115–133, which correspond to the core region of CgeA that was well resolved in the cryo-EM map. The cryo-EM structure closely matches the C-terminal region of the AlphaFold 3-predicted structure, as shown by the superposition of the AlphaFold 3 model onto the cryo-EM map (Fig. 3C).

The CgeA hexamer consists of six identical subunits organized in a ring-like arrangement, resembling a six-spoked wagon wheel. Despite the point mutations designed to form disulfide bond-anchoring helices to the inner core domains, the resulting cryo-EM map primarily captured the core hexameric structure, which was clearly resolved in the cryo-EM map (Fig. 3C). Although the introduction of cysteine mutations improved the overall image quality of the cryo-EM reconstruction, the disulfide bond itself, presumably formed by the engineered cysteine residues, could not be unambiguously visualized in the final cryo-EM map.

### Structural analysis of the CgeA hexamer

**Fig. 4.**
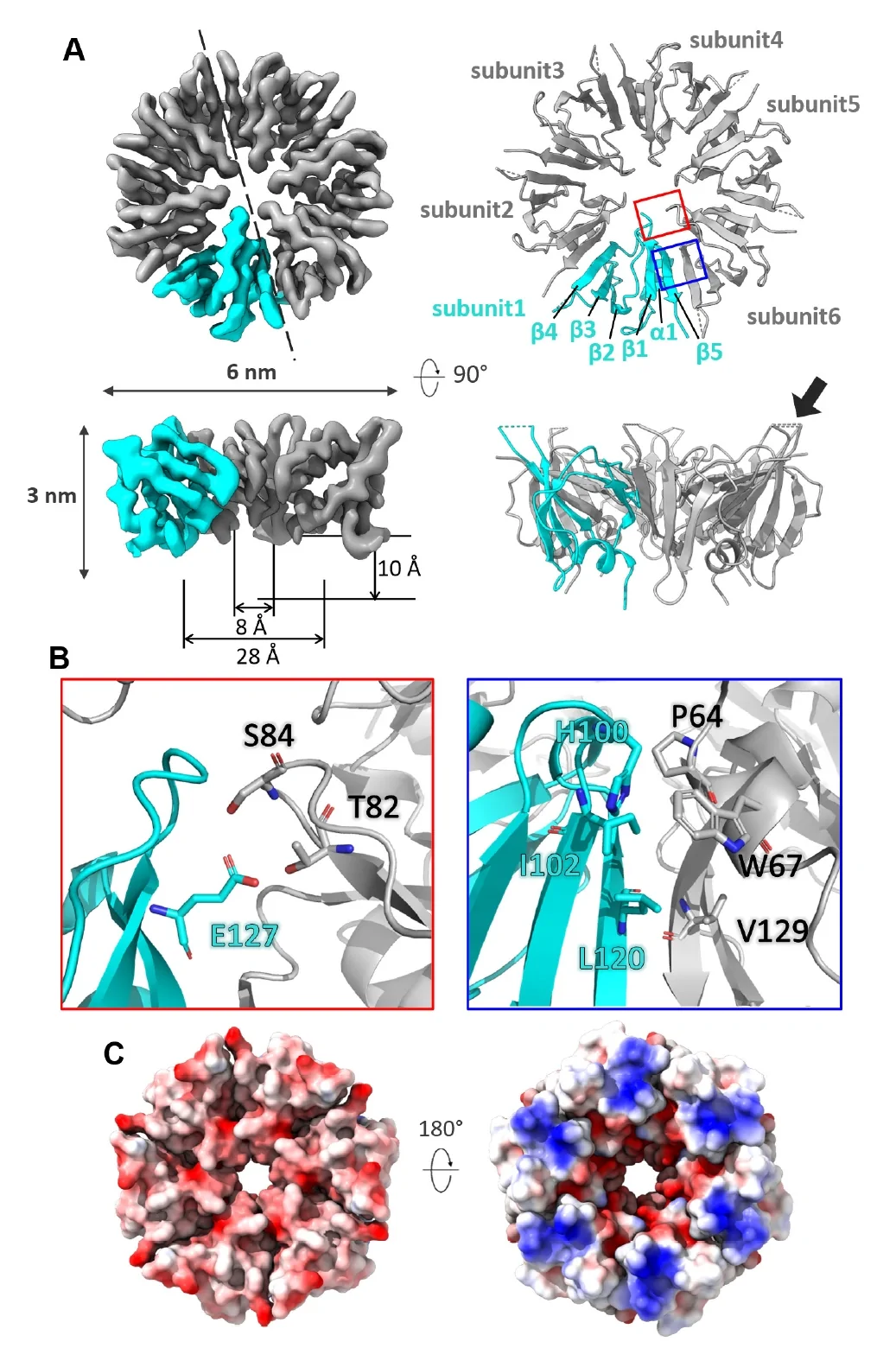
Structural analysis of CgeA protein. (A) Electron microscopy map (left) and atomic model (right) of the CgeA hexamer. One subunit in the hexamer is shown in cyan, while the other subunits are displayed in gray. The dashed line in the top left panel indicates the direction of the cross-sectional view presented in the bottom left panel. The boxed regions highlight areas that are shown in greater detail in (B). In the bottom right panel, the flexible loop region (residues 110–114) is not modeled and shown as a dashed line. A black arrow indicates this loop, where Thr112 is expected to position. (B) Close-up views of key interactions in the CgeA hexamer. The left panel in the red box highlights residues involved in hydrophilic interactions around the central pore. The right panel in the blue box shows residues contributing to hydrophobic interactions at the subunit interface. (C) Electrostatic surface representation of CgeA. The left panel shows the surface facing the glycosylation site, while the right panel represents the surface oriented toward the spore core. The hexagons indicate putative glycosyl moiety attached at Thr112.

The C-terminal domain of each subunit in the CgeA hexamer comprises a single α-helix, five β-strands, and loops that connect these secondary structures (Fig. 4A). The cryo-EM density map reveals a well-defined structure with a width of approximately 6 nm and a height of 3 nm. The hexamer forms a well-organized central pore, which begins with a wide opening at the top, measuring approximately 28 Å in diameter, and gradually narrows to a consistent 8 Å near the middle. The pore has a depth of approximately 10 Å, exhibiting a funnel-like shape.

Around the central pore, a hydrogen-bonding network is established between residues, such as Ser84 and Thr82 from one subunit and Glu127 from the adjacent subunit, further stabilizing the inter-subunit interface. The hydrophilic surface of the central pore suggests a potential role in mediating interactions with other crustal proteins or serving as a binding site for enzymes involved in glycosylation. The interfaces between adjacent subunits are predominantly stabilized by hydrophobic interactions and π-π stacking. Key residues contributing to these interactions include Leu120, Ile102, and His100 from one subunit and Val129, Pro64, and Trp67 from the neighboring subunit (Fig. 4B).

The glycosylated Thr112 residue likely plays a crucial role in the function of CgeA within the spore crust of *B. subtilis* (Nakaya et al., 2023). In the cryo-EM structure at 3.05 Å resolution, the core region of the CgeA hexamer is well resolved, while the loop encompassing residues 110–114 is disordered and not visible in the density map. However, the surrounding residues, including 109 and 115, are defined in the map and appear to lie on the solvent-exposed surface, suggesting that Thr112 is likely positioned in the same region near the rim of the central pore (Fig. 3C).

Although the loop containing Thr112 is not resolved in the structure, its presumed location suggests that the glycosylated part may extend outward from the spore surface, potentially mediating interactions with the external environment or other crustal proteins. Notably, the glycosylation face of CgeA is predominantly negatively charged, providing a hydrophilic and negatively charged surface with glycosylation chains on the outermost layer of the spores (Fig. 4C, left). In contrast, the opposite face of the hexamer is characterized by a junction with flexible N-terminal helices and slightly concave features around the central hole (Fig. 4C, right). This face is oriented toward the spore core. Flexible N-terminal helices and their inward-facing surfaces may facilitate interactions with other spore components.

### Predicted model of CgeB in complex with UDP-glucose

CgeB is a putative glycosyltransferase encoded in the *cgeA*B operon (Roels and Losick, 1995). Based on its genomic context and predicted functional domains, we hypothesized that it may be involved in the glycosylation of CgeA. Multiple sequence alignment analysis has classified CgeB as a member of the UDP-glycosyltransferase superfamily. To predict its structure, the local version of AlphaFold 3 (Abramson et al., 2024; Park et al., 2025) was used with the CgeB sequence and the ligand UDP-glucose.

**Fig. 5.**
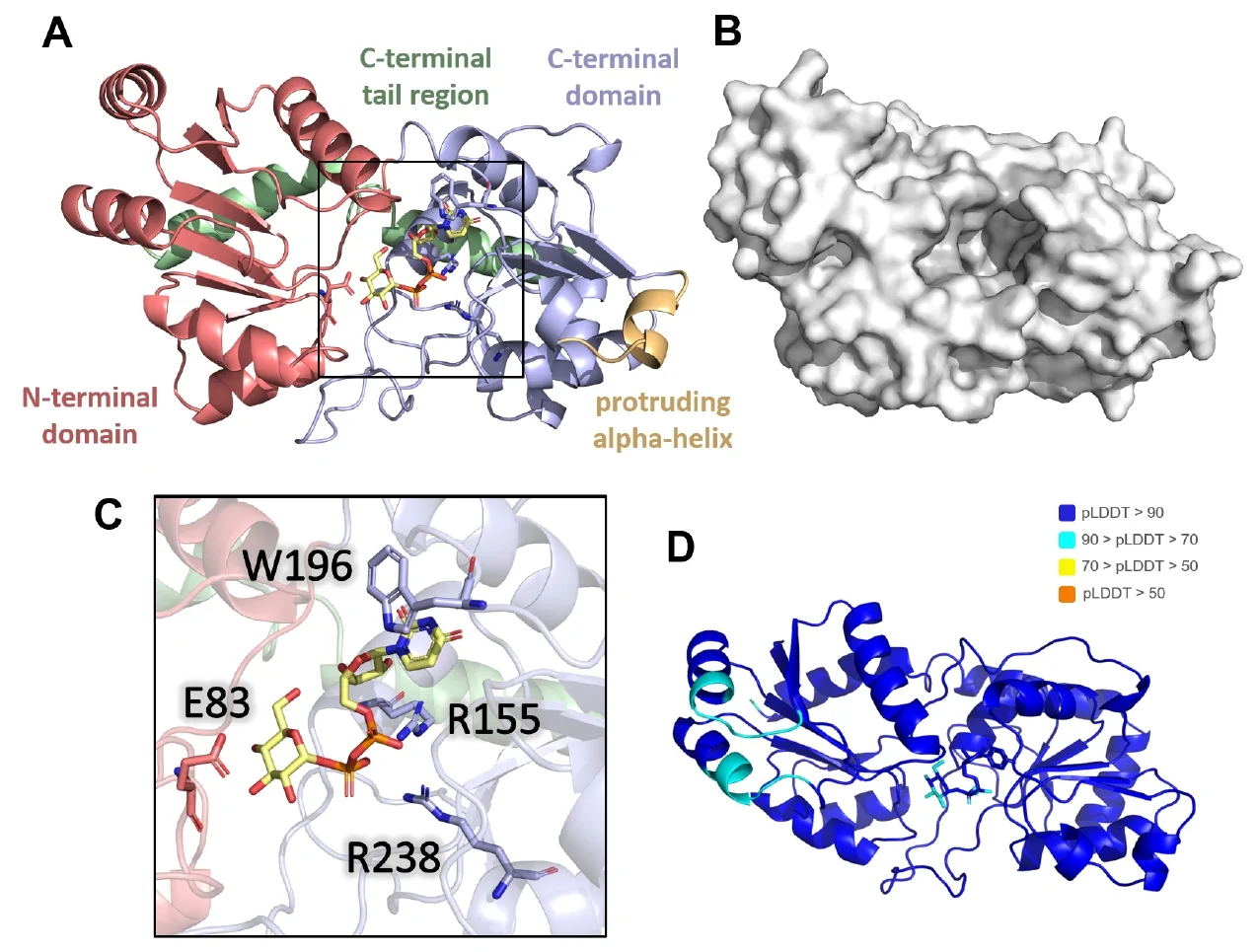
Predicted molecular model of CgeB in complex with UDP-glucose. (A) Domain organization of CgeB. The N-terminal domain is shown in pink, C-terminal domain in light purple, and C-terminal α-helical tail region in green. The protruding α-helix in the C-terminal domain is highlighted in yellow. The boxed region indicates the area shown in greater detail in (C). (B) Surface representation of the AlphaFold 3-predicted structure of CgeB, revealing a prominent pocket between the N- and C-terminal domains. (C) A close-up view of the UDP-glucose binding, with key residues involved in binding displayed as stick. (D) Cartoon representation of the AlphaFold 3-predicted model, colored by pLDDT scores to illustrate structural reliability.

The predicted AlphaFold 3 model revealed three distinct domains: an N-terminal domain (residues 1–128), C-terminal domain (residues 129–281), and C-terminal alpha-helical tail region (residues 282–317) (Fig. 5A). A prominent pocket was observed at the front between the N- and C-terminal domains (Fig. 5B). Notably, a protruding alpha helix (residues 180–188) was identified in the C-terminal domain. The UDP-glucose molecule is positioned within the binding pocket. The model demonstrated a high-confidence binding mode for UDP-glucose, as supported by pLDDT and iPTM scores (Figs. 5D and S5A).

Key interactions within the predicted binding pocket were identified. Trp196 stabilized the uridine base of the UDP moiety through π-π interactions, while Arg155 and Arg238 neutralized the negative charges of the diphosphate group (Fig. 5C). Additionally, Glu83, positioned in a loop above the glucose moiety, was predicted to function as a catalytic residue facilitating the transfer of the glycosyl group to the target site on the substrate protein. These four key residues were conserved across CgeB homologs in various *Bacillus* species, supporting the plausibility of their functional roles in glycosyl transfer (Fig. S5B).

Further modeling using UDP-galactose, UDP-N-acetylglucosamine (UDP-GlcNAc), and UDP-xylose yielded similar binding configurations (Fig. S5A). These findings suggest that the UDP-monosaccharide likely serves as a co-substrate for CgeB, although the specific UDP-monosaccharide remains unidentified.

### UDP-glucose-dependent glycosyltransferase activity of CgeB toward CgeA

**Fig. 6.**
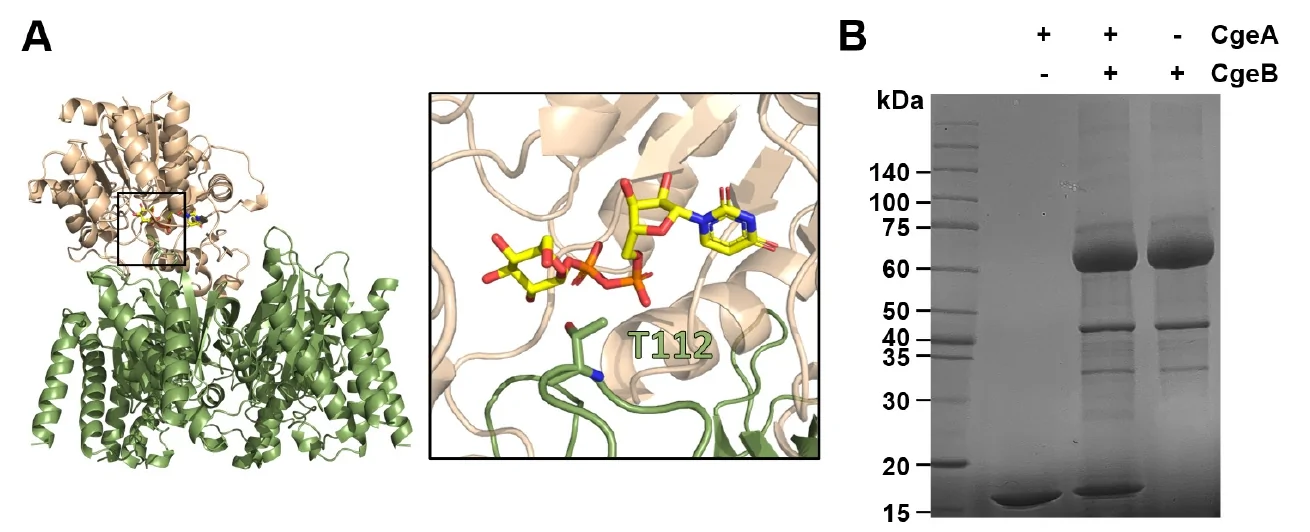
Glycosyltransferase activity of CgeB toward CgeA. (A) AlphaFold 3-predicted model of the CgeA (green) - CgeB (light orange) complex bound to UDP-glucose. The left panel shows the overall complex, with the boxed region indicating the UDP-glucose binding site. The right panel provides a close-up view of key residues involved in UDP-glucose interaction. CgeA Thr112 residue is shown in the stick representation. (B) Sodium dodecyl sulfate polyacrylamide gel electrophoresis (SDS-PAGE) analysis of glycosylation of CgeA by CgeB. CgeA was incubated alone or with CgeB and UDP-glucose at 37°C for 18 h. The reaction products were analyzed by SDS-PAGE. The band corresponding to CgeA shows a slight upward shift in the presence of CgeB and UDP-glucose, indicating glycosylation.

To examine the interaction between CgeA and CgeB, we conducted a glycosylation assay using UDP-glucose as a donor substrate. After prolonged incubation, a slight upward shift in the CgeA protein band was observed by SDS–PAGE (Fig. 6), suggesting that CgeA undergoes glycosylation mediated by CgeB. These findings imply that CgeB can transfer glucose to CgeA using UDP-glucose; however, the low activity observed warrants further investigation to confirm the specificity of this modification and to identify the physiological UDP-monosaccharide substrate.

To gain structural insight into this interaction, we utilized AlphaFold 3 to predict a ternary complex consisting of CgeA, CgeB, and UDP-glucose. In the predicted complex (Fig. S6), CgeB is modeled to bind UDP-glucose and associate with the hexameric assembly of CgeA. A protruding loop from the C-terminal domain of CgeB extends into the central pore of the CgeA hexamer, placing the putative active site of CgeB in close proximity to Thr112 of CgeA—the presumed glycosylation site.

## Discussion

In this study, we structurally and functionally characterized CgeA, providing key insights into its role in assembling, glycosylating, and enhancing the adaptability of *B. subtilis* spore crust. By resolving the cryo-EM structure of the core region of CgeA at 3.05 Å resolution, we elucidated its homohexameric assembly, which adopts a distinctive doughnut-like shape. This hexameric organization presents functionally distinct surfaces. Although the loop containing Thr112 (residues 110–114) was not resolved in the density map, its position can be reasonably inferred from the surrounding modeled residues, including 109 and 115, which lie on the exposed face of the hexamer. This structural context suggests that Thr112 resides in an outward-facing, flexible loop, consistent with its proposed role as a glycosylation site. The spatial arrangement supports the hypothesis that Thr112 is positioned appropriately for modification by the putative glycosyltransferase CgeB.

AlphaFold 3-based modeling suggests that CgeB functions as a UDP-glucose- (or other UDP-monosaccharide)-dependent glycosyltransferase. Further modeling and preliminary biochemical analysis using the CgeA hexamer and monomer supported the specific transfer reaction of CgeB to Thr112 on CgeA, highlighting the molecular basis for CgeA glycosylation within the spore crust.

Modeling studies of CgeB and CgeA suggest that CgeB can transfer only a single glycosyl group to CgeA. However, an extended glycosylation chain at Thr112 likely increases the hydrophilicity of the spore surface, facilitating its dispersal in aqueous environments. Therefore, we propose that another glycosyltransferase may be involved in further elongation of the glycan chain. One candidate is CgeD, encoded by the *cgeCDE* operon (distinct from the *cgeAB* operon), which has been implicated in exopolysaccharide synthesis during sporulation (Roels and Losick, 1995; Shuster et al., 2019b). While CgeD may contribute to this maturation step, we acknowledge that other sporulation-related glycosyltransferases—such as YfnH, SpsI, or YtdA—could also be involved (Dubois et al., 2020; Shuster et al., 2019b). Further experimental validation is required to determine the specific roles of these enzymes. These modifications are crucial for reducing crustal hydrophobicity and promoting spore dispersal, ultimately contributing to the ecological success of *B. subtilis*. Furthermore, the predominantly negatively charged glycosylated face may help maintain a hydrated, non-adhesive surface, preventing excessive aggregation of spores in moist environments.

**Fig. 7.**
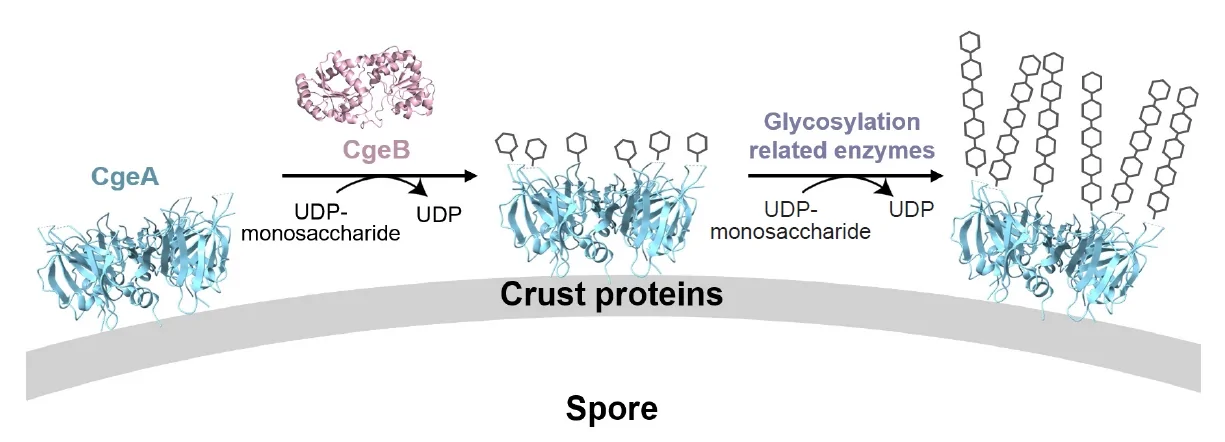
Model for the glycosylation of CgeA during spore crust assembly. The cryo-EM structure of CgeA (cyan) and AlphaFold 3 model of CgeB (pink) are shown. CgeB is proposed to transfer a single glycosyl group to CgeA using a UDP-monosaccharide donor. Further extension may involve other glycosylation-related enzymes. Glycosylated CgeA may interact with crust proteins such as CotY and CotZ (Krajčíková et al., 2017). Glycan chains are shown schematically.

The opposite face of the CgeA hexamer, marked by its junction with flexible N-terminal helices, is structurally and functionally distinct. This surface may interact with external molecules or other crust proteins, such as CotY and CotZ, during crust assembly. Supporting this, yeast two-hybrid studies have shown that CgeA interacts with the crust morphogenetic protein CotY (Krajčíková et al., 2017), supporting its role in crust organization. Based on these findings, we hypothesize that glycosylated CgeA may be anchored to the outermost layer of the crust through protein–protein interactions, while undergoing initial glycan modification by CgeB. Additional glycosyltransferases, such as CgeD, may subsequently extend the glycan chain, contributing to the full maturation of the glycosylated structure (Fig. 7). Together, these insights provide a structural basis for understanding how CgeA glycosylation modulates spore surface hydrophilicity.

The findings of this study have important implications beyond microbial ecology, particularly for food microbiology and food safety. *Bacillus* spores present a persistent challenge in the food industry because of their high resistance to heat, desiccation, and chemical disinfectants. In processing environments, spores can adhere to food contact surfaces, leading to contamination risks and biofilm formation, which complicate sanitation procedures. Given that the hydrophilicity of the spore surface affects its ability to adhere to surfaces and disperse in aqueous environments, CgeA-mediated glycosylation may play a crucial role in determining the persistence of *B. subtilis* spores in food-processing facilities.

*B. subtilis* is widely used as a probiotic and fermentation starter in food production, making its spore formation and dispersal mechanisms highly relevant to food biotechnology. Glycosylation of CgeA may influence how probiotic *B. subtilis* strains colonize specific environments, interact with the host microbiota, or remain stable in food products. Understanding the glycosylation process can inform strategies for modulating spore properties for improved probiotic formulations, enhanced food preservation, and targeted microbial control approaches in food manufacturing.

## Supplementary Information

Table S1. Nucleotide sequences of the primers used in this study

Table S2. Cryo-electron microscopy data collection and processing statistics

Fig. S1. Cryo-electron microscopy (Cryo-EM) processing workflow for CgeA.

Fig. S2. Detailed information of the CgeA AlphaFold 3-predicted model. (A) The left panels display the predicted model colored by pLDDT scores, while the right panel shows the predicted alignment error (PAE). (B) The left panel highlights all hydrophilic residues in the N-terminal helical region as stick, while the right panel marks these residues in red within the full CgeA sequence.

Fig. S3. Size-exclusion chromatography (SEC) analysis of CgeA wild-type and A42C/D97C mutant. (A) The wild-type CgeA is represented by a red line, while the mutant is shown in blue. Peaks corresponding to different forms of CgeA are annotated with their estimated molecular weights, calculated based on the protein size reference shown in (B). (B) Calibration curve of the SEC column. The curve was generated by plotting the elution volumes of a series of reference proteins against their known molecular weights (log kDa) using a Superdex 200 Increase 16/600 pg size-exclusion chromatography column. The reference proteins were obtained from the Gel Filtration Calibration Kits (LMW, HMW) (Cytiva).

Fig. S4. Cryo-electron microscopy (Cryo-EM) data processing. (A) Cryo-EM processing workflow for CgeA A42C/D97C. (B) Local resolution maps calculated using CryoSPARC. The maps are color-coded to represent varying resolutions, from red (2.7 Å) to blue (3.3 Å). (C) Global resolution estimates from gold-standard refinement, with a calculated resolution of 3.05 Å based on the Fourier shell correlation (FSC) 0.143 cut-off. (D) Model-to-map resolution estimate (FSC_model) with a 0.5 cutoff, indicating a resolution of 4.0 Å, calculated using real space refinement in Phenix (Adams et al., 2010).

Fig. S5. Structural prediction and sequence conservation analysis of CgeB. (A) AlphaFold 3-predicted models of CgeB bound to various UDP-monosaccharides. Each model is colored based on pLDDT scores, with the corresponding predicted alignment error (PAE) maps shown on the right. (B) Alignment of CgeB sequences from related *Bacillus* species using the Clustal algorithm. Identical amino acids across all strains are shaded in black, while residues conserved in more than 70% of the strains are shaded in gray. Key residues predicted to be involved in UDP-glucose binding are highlighted with a red box.

Fig. S6. Detailed information of the CgeA-CgeB AlphaFold 3-predicted model. AlphaFold 3-predicted model of the CgeA-CgeB complex bound to UDP-glucose. The left panels display the predicted model colored by the pLDDT scores, whereas the right panel shows the predicted alignment error (PAE).
