# Supplementary material for: Cryo-EM structure of the glycosylated protein CgeA in the crust of Bacillus subtilis endospores
Source: J Microbiol. 2025 Oct 31;63(10):e2504013. doi: 10.71150/jm.2504013 (PMC13577073; doi:10.71150/jm.2504013)
Supplement: Supplementary file 1 [file jm-2504013-Supplementary-Fig-S1.pdf]

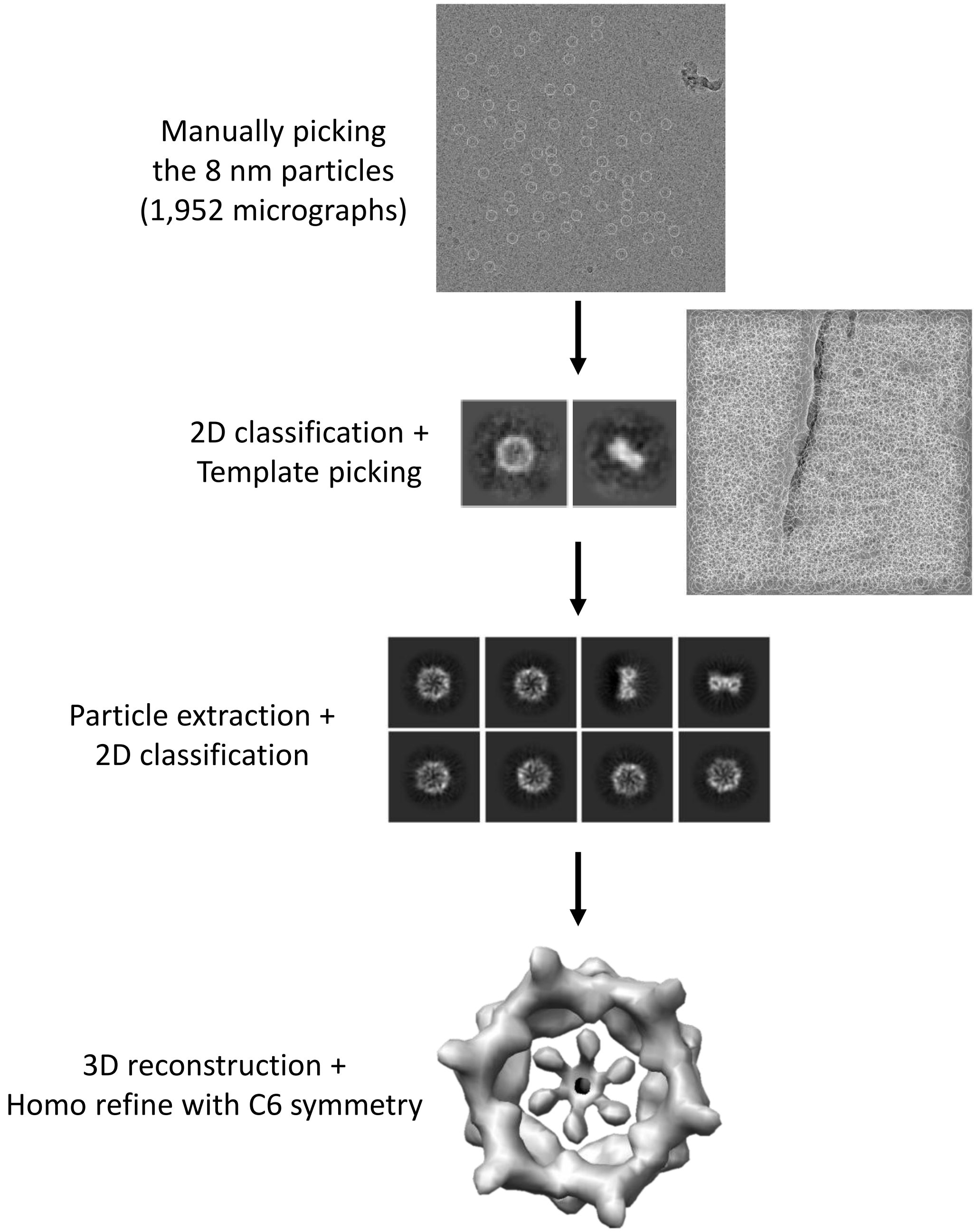
Manually picking
the 8 nm particles
(1,952 micrographs)
2D classification +
Template picking
Particle extraction +
2D classification
3D reconstruction +
Homo refine with C6 symmetry
