# Supplementary material for: Cryo-EM structure of the glycosylated protein CgeA in the crust of Bacillus subtilis endospores
Source: J Microbiol. 2025 Oct 31;63(10):e2504013. doi: 10.71150/jm.2504013 (PMC13577073; doi:10.71150/jm.2504013)
Supplement: Supplementary file 2 [file jm-2504013-Supplementary-Fig-S2.pdf]

**A**

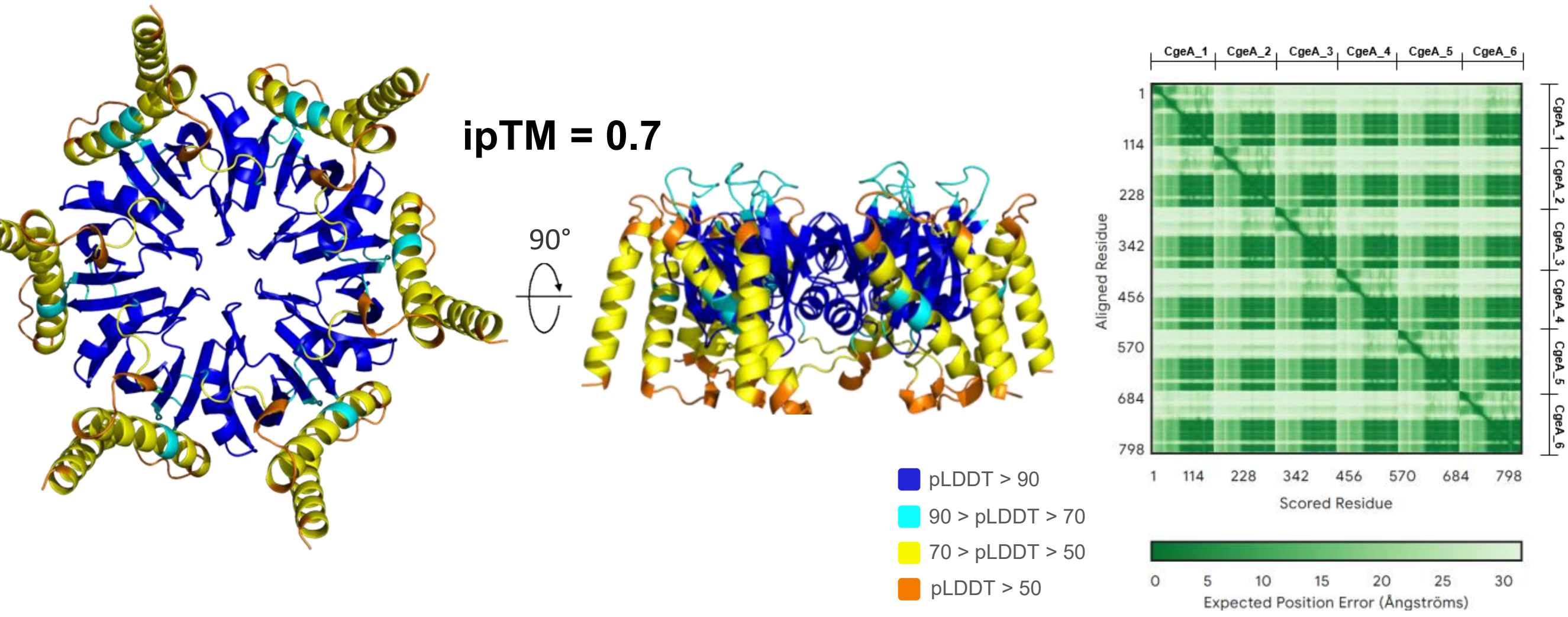


**B**

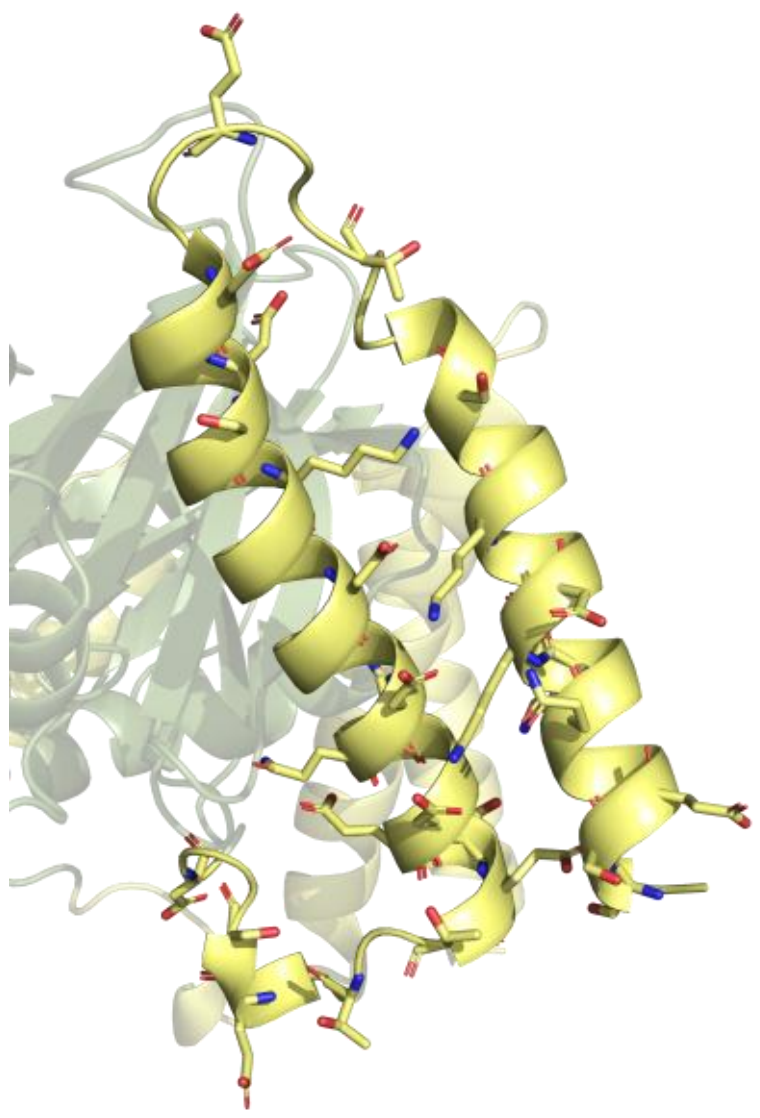

1 MSSENAQLKKDLIKAVLSPLFPTATEG
28 GENMDSNLKALLDAAIDQKVDESETVT
55 AESILDPSLPARWIFARITPGTTISIV
82 TDSGDMIGPVVFVAFDQVHGIVFVTQE
109 SSVTPAGQATTLIDVDKVESVTFFS
