# Supplementary material for: Cryo-EM structure of the glycosylated protein CgeA in the crust of Bacillus subtilis endospores
Source: J Microbiol. 2025 Oct 31;63(10):e2504013. doi: 10.71150/jm.2504013 (PMC13577073; doi:10.71150/jm.2504013)
Supplement: Supplementary file 3 [file jm-2504013-Supplementary-Fig-S3.pdf]

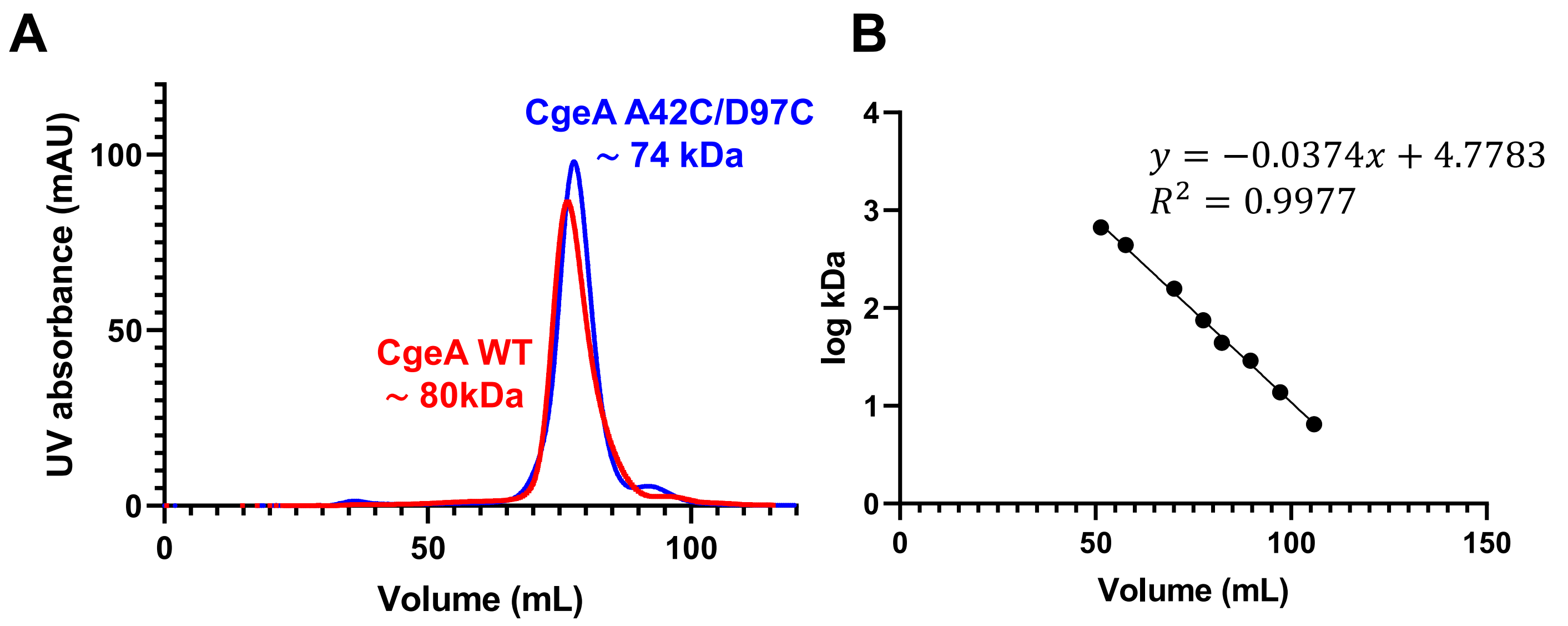
A
CgeA A42C/D97C
~ 74 kDa
CgeA WT
~ 80kDa
UV absorbance (mAU)
100
50
0
0
50
100
Volume (mL)
B
y = −0.0374x + 4.7783
R² = 0.9977
log kDa
4
3
2
1
0
0
50
100
150
Volume (mL)
