# Supplementary material for: Cryo-EM structure of the glycosylated protein CgeA in the crust of Bacillus subtilis endospores
Source: J Microbiol. 2025 Oct 31;63(10):e2504013. doi: 10.71150/jm.2504013 (PMC13577073; doi:10.71150/jm.2504013)
Supplement: Supplementary file 4 [file jm-2504013-Supplementary-Fig-S4.pdf]

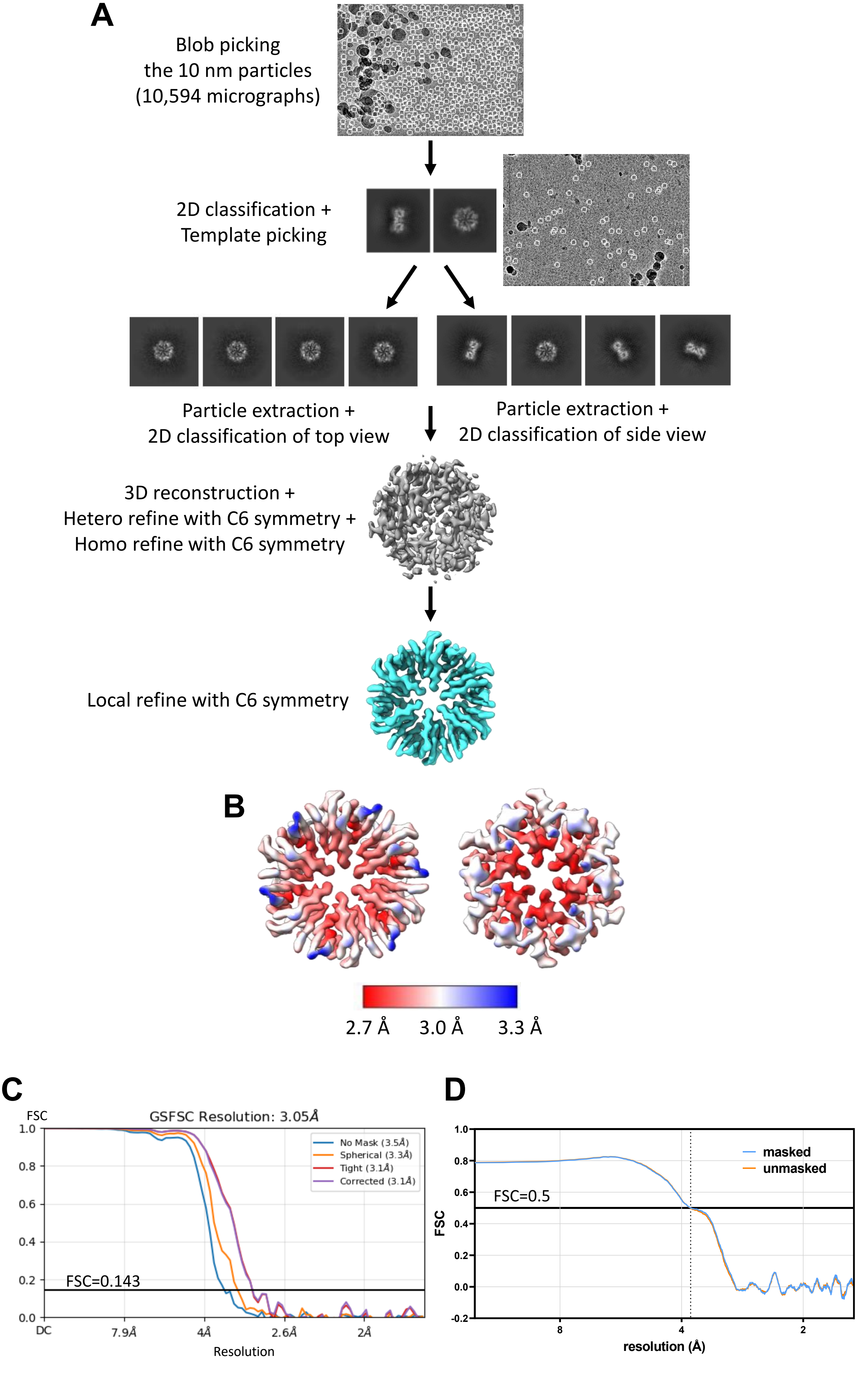

A
Blob picking
the 10 nm particles
(10,594 micrographs)
2D classification +
Template picking
Particle extraction +
2D classification of top view
Particle extraction +
2D classification of side view
3D reconstruction +
Hetero refine with C6 symmetry +
Homo refine with C6 symmetry
Local refine with C6 symmetry
B
2.7 Å
3.0 Å
3.3 Å
C
GSFSC Resolution: 3.05Å
FSC
No Mask (3.5Å)
Spherical (3.3Å)
Tight (3.1Å)
Corrected (3.1Å)
FSC=0.143
DC
7.9Å
4Å
2.6Å
2Å
Resolution
D
masked
unmasked
FSC=0.5
FSC
resolution (Å)
