# Supplementary material for: Cryo-EM structure of the glycosylated protein CgeA in the crust of Bacillus subtilis endospores
Source: J Microbiol. 2025 Oct 31;63(10):e2504013. doi: 10.71150/jm.2504013 (PMC13577073; doi:10.71150/jm.2504013)
Supplement: Supplementary file 5 [file jm-2504013-Supplementary-Fig-S5.pdf]

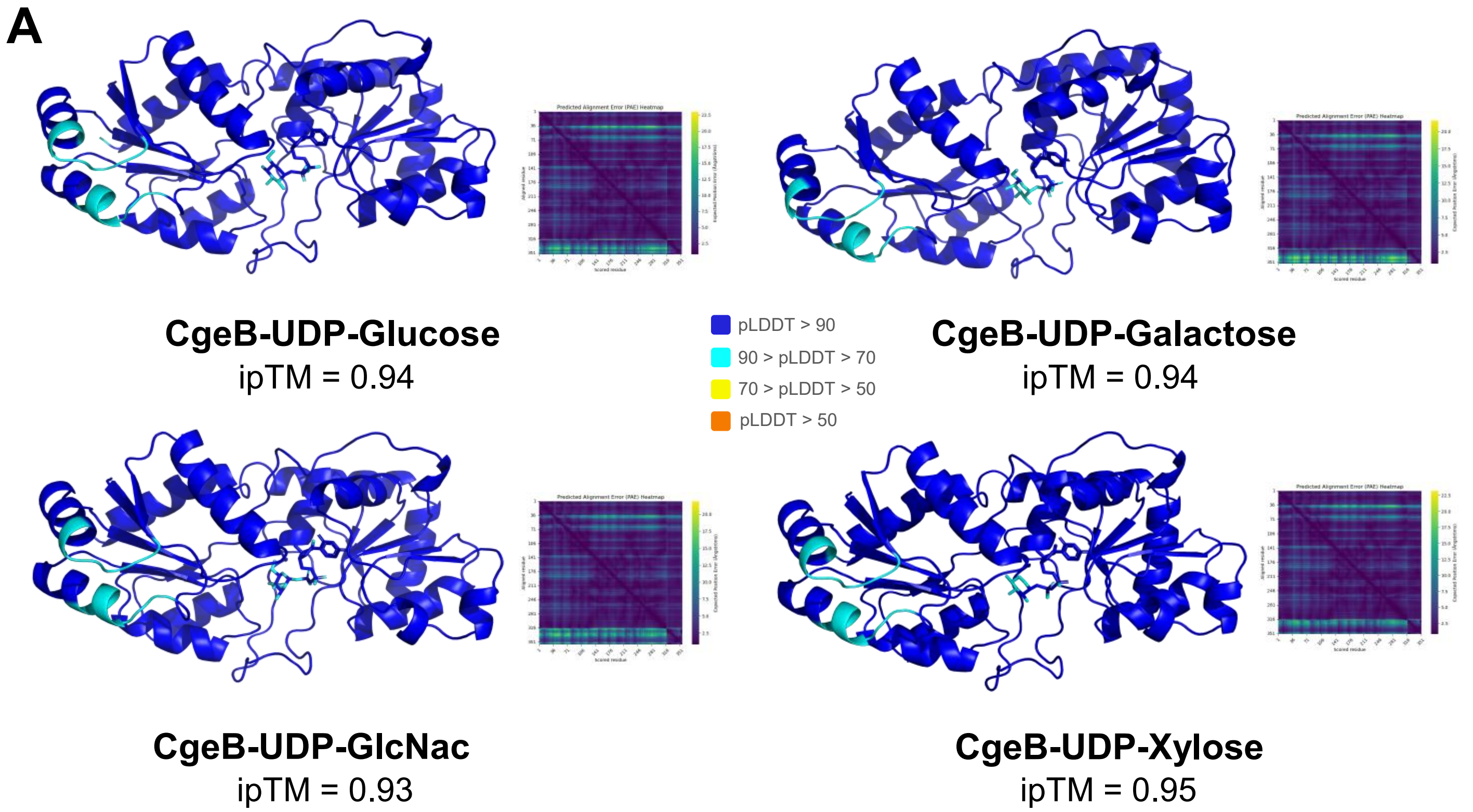
A
pLDDT > 90
90 > pLDDT > 70
70 > pLDDT > 50
pLDDT > 50
CgeB-UDP-Glucose
ipTM = 0.94
CgeB-UDP-Galactose
ipTM = 0.94
CgeB-UDP-GlcNac
ipTM = 0.93
CgeB-UDP-Xylose
ipTM = 0.95

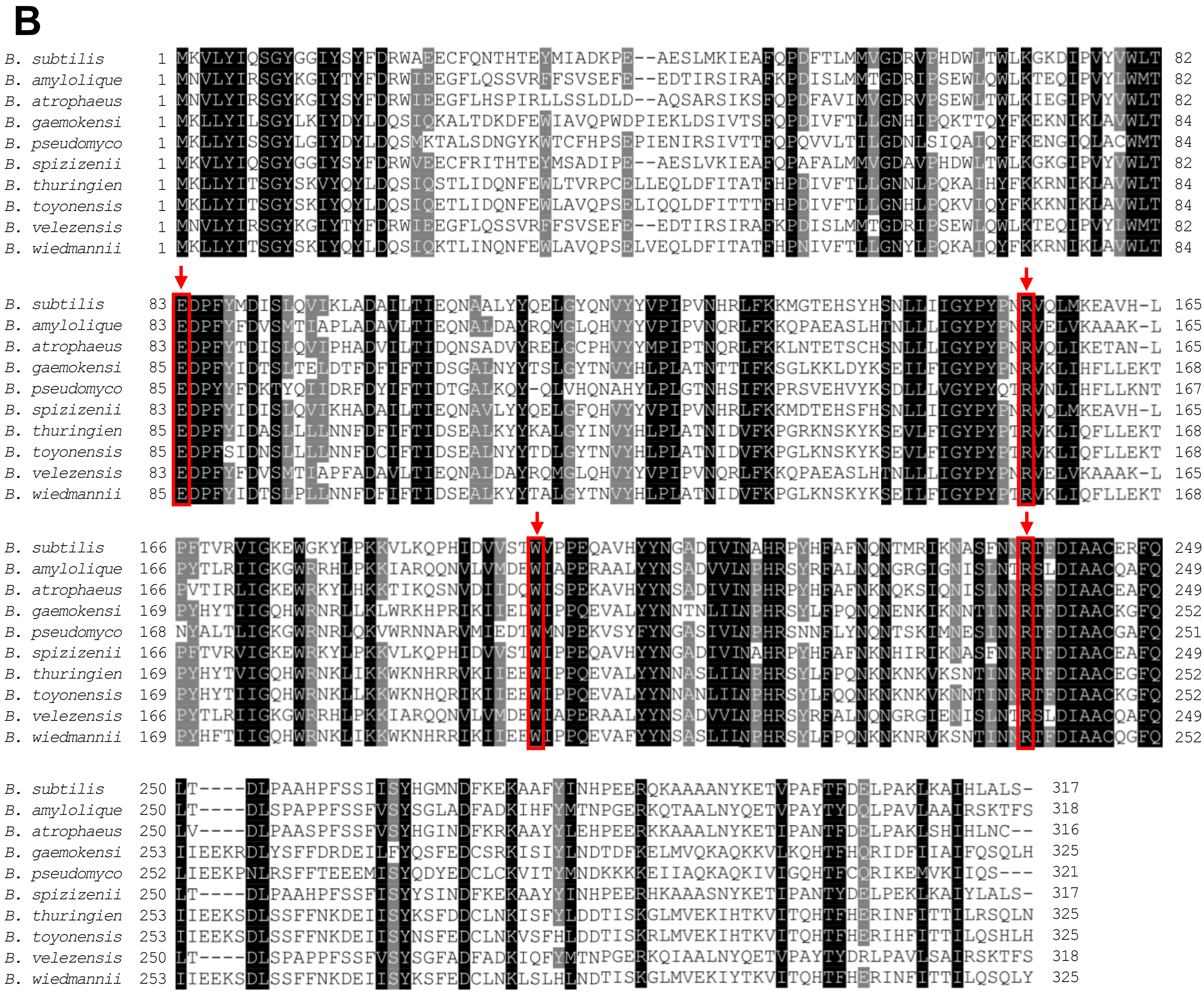
B
B. subtilis 1 MKVLYIQSGYGGIYSYFDRWAEECFQNTHTEYMIADKPE--AESLMKIEAFQPDFTLMMVGDRVPHDWLTWLKGKDIPVYVWLT 82
B. amylolique 1 MNVLYIRSGYKGIYTYFDRWIEEGFLQSSVRFFSVSEFE--EDTIRSIRAFKPDISLMMTGDRIPSEWLQWLKTEQIPVYLWMT 82
B. atrophaeus 1 MNVLYIRSGYKGIYSYFDRWIEEGFLHSPIRLLSSLDLD--AQSARSIKSFQPDFAVIMVGDRVPSEWLTWLKIEGIPVYVWLT 82
B. gaemokensi 1 MKLLYILSGYLKIYDYLDQSIQKALTDKDFEWIAVQPWDPIEKLDSIVTSFQPDIVFTLLGNHIPQKTTQYFKEKNIKLAVWLT 84
B. pseudomyco 1 MKLLYISSGYLGIYDYLDQSMKTALSDNGYKWTCFHPSEPIENIRSIVTTFQPQVVLTILGDNLSIQAIQYFKENGIQLACWMT 84
B. spizizenii 1 MKVLYIQSGYGGIYSYFDRWVEECFRITHTEYMSADIPE--AESLVKIEAFQPAFALMMVGDAVPHDWLTWLKGKGIPVYVWLT 82
B. thuringien 1 MKLLYITSGYSKVYQYLDQSIQSTLIDQNFEWLTVRPCELLEQLDFITATFHPDIVFTLLGNNLPQKAIHYFKKRNIKLAVWLT 84
B. toyonensis 1 MKLLYITSGYSKIYQYLDQSIQETLIDQNFEWLAVQPSELIQQLDFITTTFHPDIVFTLLGNHLPQKVIQYFKKKNIKLAVWLT 84
B. velezensis 1 MNVLYIRSGYKGIYTYFDRWIEEGFLQSSVRFFSVSEFE--EDTIRSIRAFKPDISLMMTGDRIPSEWLQWLKTEQIPVYLWMT 82
B. wiedmannii 1 MKLLYITSGYSKIYQYLDQSIQKTLINQNFEWLAVQPSELVEQLDFITATFHPNIVFTLLGNYLPQKAIQYFKKRNIKLAVWLT 84
B. subtilis 83 EDPFYMDISLQVIKLADAILTIEQNAALYQELGYQNVYYVPIPVNHRLFKKMGTEHSYHSNLLIIGYPYPNRVQLMKEAVH-L 165
B. amylolique 83 EDPFYFDVSMTIAPLADAVLTIEQNALDAYRQMGLQHVYYVPIPVNQRLFKKQPAEASLHTNLLLIGYPYPNRVELVKAAAK-L 165
B. atrophaeus 83 EDPFYTDISLQVIPHADVILTIDQNSADVYRELGCPHVYYMPIPTNQRLFKKLNTETSCHSNLLLIGYPYPNRVQLIKETAN-L 165
B. gaemokensi 85 EDPFYIDTSLTELDTFDFIFTIDSGAINYYTSLGYTNVYHLPLATNTTIFKSGLKKLDYKSEILFIGYPYPNRVKLIHFLLEKT 168
B. pseudomyco 85 EDPYYFDKTYQIIDRFDYIFTIDTGALKQY-QLVHQNAHYLPLGTNHSIFKPRSVEHVYKSDLLLVGYPYQTRVNLIHFLLKNT 167
B. spizizenii 83 EDPFYIDISLQVIKHADAILTIEQNAVLYQELGFQHVYYVPIPVNHRLFKKMDTEHSFHSNLLIIGYPYPNRVQLMKEAVH-L 165
B. thuringien 85 EDPFYIDASLLLLNNFDFIFTIDSEALKYYKALGYINVYHLPLATNIDVFKPGRKNSKYKSEVLFIGYPYPTRVKLIQFLLEKT 168
B. toyonensis 85 EDPFSIDNSLLLLNNFDCIFTIDSEAINYYTDLGYTNVYHLPLATNIDVFKPGLKNSKYKSEVLFIGYPYPTRVKLIQFLLEKT 168
B. velezensis 83 EDPFYFDVSMTIAPFADAVLTIEQNALDAYRQMGLQHVYYVPIPVNQRLFKKQPAEASLHTNLLLIGYPYPNRVELVKAAAK-L 165
B. wiedmannii 85 EDPFYIDTSLPLLNNFDFIFTIDSEALKYYTALGYTNVYHLPLATNIDVFKPGLKNSKYKSEIILFIGYPYPTRVKLIQFLLEKT 168
B. subtilis 166 PFTVRVIGKEWGKYLPKKVLKQPHIDVVSTWVPPEQAVHYYNGADIVINAHRPYHFAFNQNTMRIKNASFNNRTFDIAACERFQ 249
B. amylolique 166 PYTLRIIGKGWRRHLPKKIARQQNVLVMDEWIAPERAALYYNSADVVINPHRSYRFALNQNGRGIGNISLNTRSLDIAACQAFQ 249
B. atrophaeus 166 PVTIRLIGKEWRKYLHKKTIKQSNVDIIDQWISPEKAVHYYNSADIVINPHRPYHFAFNKNQKSIQNISLNNRSFDIAACEAFQ 249
B. gaemokensi 169 PYHYTIIGQHWRNRLLKLWRKHPRIKIIEDWIPPQEVALYYNNTNLIINPHRSYLFPQNQNENKIKNNTINNRTFDIAACKGFQ 252
B. pseudomyco 168 NYALTLIGKGWRNRLQKVWRNNARVMIEDTWMNPEKVSYFYNGASIVINPHRSNNFLYNQNTSKIMNESINNRTFDIAACGAFQ 251
B. spizizenii 166 PFTVRVIGKEWRKYLPKKVLKQPHIDVVSTWIPPEQAVHYYNGADIVINAHRPYHFAFNKNHIRIKNASFNNRTFDIAACEAFQ 249
B. thuringien 169 PYHYTVIGQHWRNKLIKKWKNHRRVKIIEEWIPPQEVALYYNNASLIINPHRSYLFPQNKNKNKVKSNTINNRTFDIAACEGFQ 252
B. toyonensis 169 PYHYTIIGQHWRNKLLKKWKNHQRIKIIEEWIPPQEVALYYNNANLIINPHRSYLFQQNKNKNKVKNNTINNRTFDIAACKGFQ 252
B. velezensis 166 PYTLRIIGKGWRRHLPKKIARQQNVLVMDEWIAPERAALYYNSADVVINPHRSYRFALNQNGRGIENISLNTRSLDIAACQAFQ 249
B. wiedmannii 169 PYHFTIIGQHWRNKLIKKWKNHRRIKIIEEWIPPQEVAFYYNSASLIINPHRSYLFPQNKNKNRVKSNTINNRTFDIAACQGFQ 252
B. subtilis 250 LT----DLPAAHPFSSIISYHGMNDFKEKAAFYINHPEERQKAAAANYKETVPAFTFDELPAKLKAIHLALS- 317
B. amylolique 250 LT----DLSPAPPFSSFVSYSGLADFADKIHFYMTNPGERKQTAALNYQETVPAYTYDQLPAVLAAIRSKTFS 318
B. atrophaeus 250 LV----DLPAASPFSSFVSYHGINDFKRKAAYYLEHPEERKKAAALNYKETIPANTFDELPAKLSHIHLNC-- 316
B. gaemokensi 253 IIEEKRDLYSFFDRDEILFYQSFEDCSRKISIYLNDTDFKELMVQKAQKKVLKQHTFHQRIDFIIAIFQSQLH 325
B. pseudomyco 252 LIEEKPNLRSFFTEEEMISYQDYEDCLCKVITYMNDKKKEIIAQKAQKIVIGQHTFCQRIKEMVKIIQS--- 321
B. spizizenii 250 LT----DLPAAHPFSSFISYYSINDFKEKAAYYINHPEERHKAAASNYKETIPANTYDELPEKLKAIYLALS- 317
B. thuringien 253 IIEEKSDLSSFFNKDEIISYKSFDDCLNKISFYLDDTISKGLMVEKIHTKVITQHTFHERINFITTILRSQLN 325
B. toyonensis 253 IIEEKSDLSSFFNKDEIISYNSFEDCLNKVSFHLDDTISKRLMVEKIHTKVITQHTFHERIHFITTILQSHLH 325
B. velezensis 250 LT----DLSPAPPFSSFVSYSGFADFADKIQFYMTNPGERKQIAALNYQETVPAYTYDRLPAVLSAIRSKTFS 318
B. wiedmannii 253 IIEEKSDLSSFFNKDEIISYKSFEDCLNKLSLHLNDTISKGLMVEKIYTKVITQHTFHERINFITTILQSQLY 325
