# Supplementary material for: Cryo-EM structure of the glycosylated protein CgeA in the crust of Bacillus subtilis endospores
Source: J Microbiol. 2025 Oct 31;63(10):e2504013. doi: 10.71150/jm.2504013 (PMC13577073; doi:10.71150/jm.2504013)
Supplement: Supplementary file 6 [file jm-2504013-Supplementary-Fig-S6.pdf]

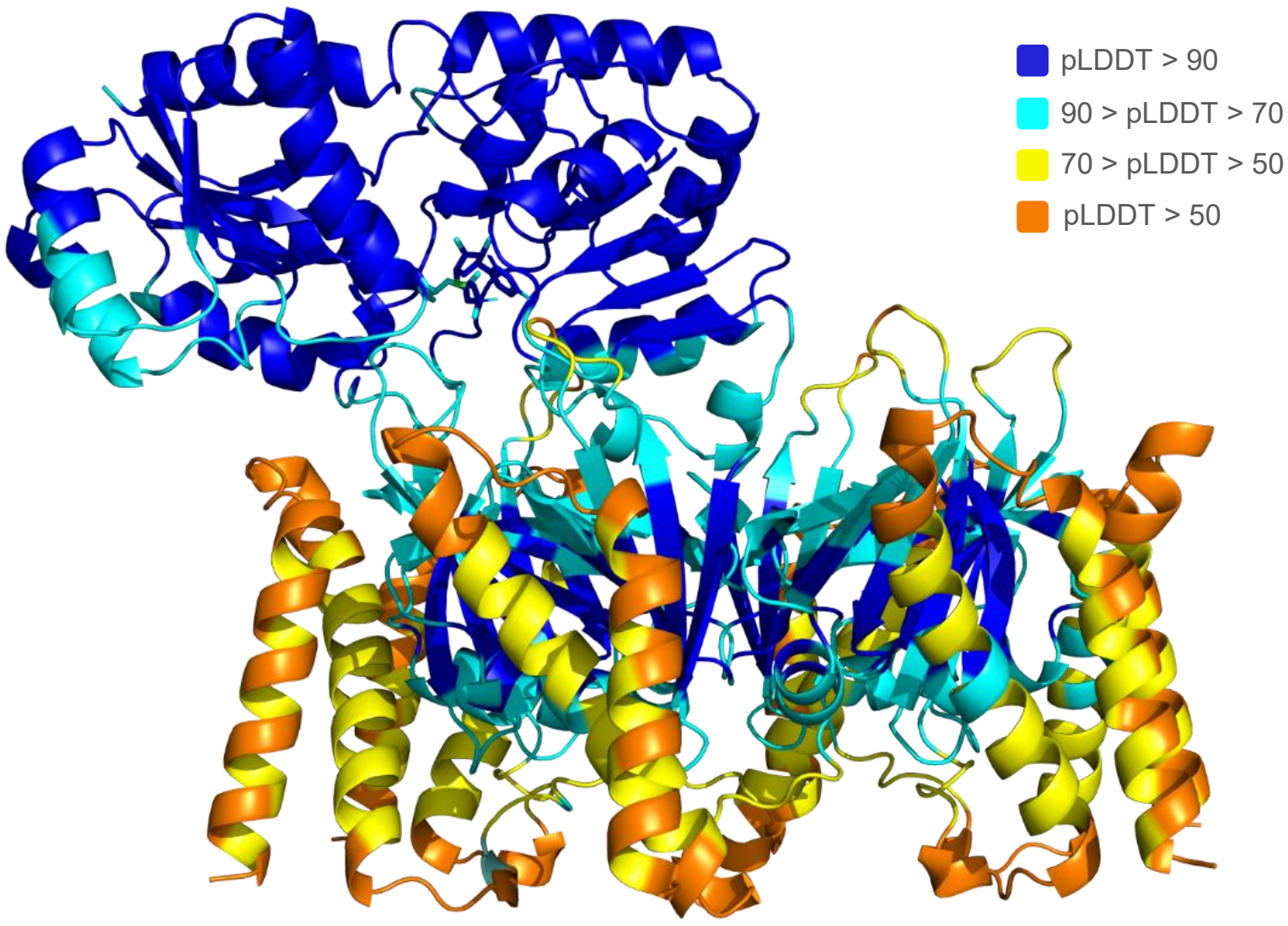
pLDDT > 90
90 > pLDDT > 70
70 > pLDDT > 50
pLDDT > 50

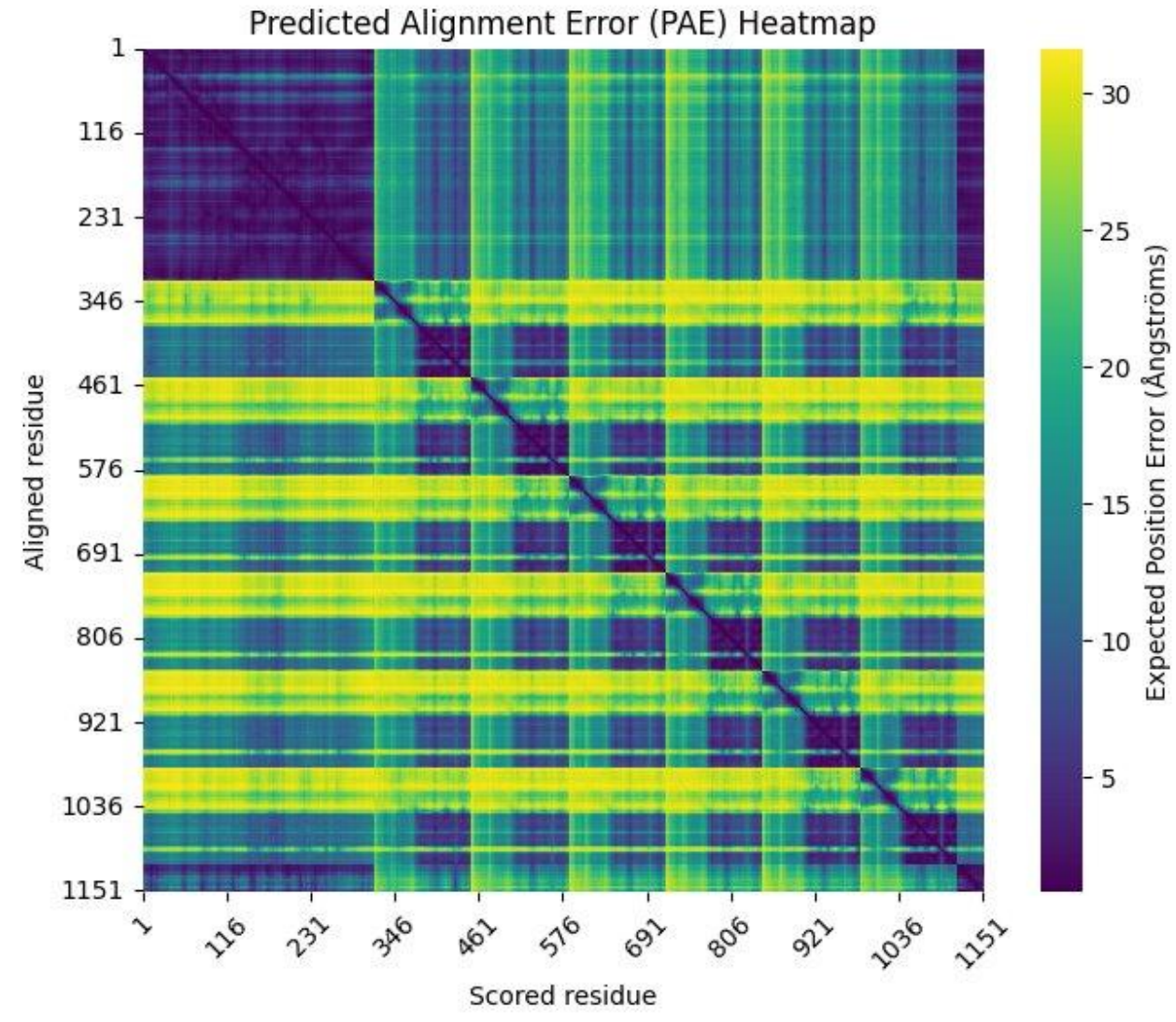
Predicted Alignment Error (PAE) Heatmap
Aligned residue
Scored residue
Expected Position Error (Ångströms)
1
116
231
346
461
576
691
806
921
1036
1151
30
25
20
15
10
5
