# Supplementary material for: Cryo-EM structure of the glycosylated protein CgeA in the crust of Bacillus subtilis endospores
Source: J Microbiol. 2025 Oct 31;63(10):e2504013. doi: 10.71150/jm.2504013 (PMC13577073; doi:10.71150/jm.2504013)
Supplement: Supplementary file 7 [file jm-2504013-Supplementary-Table-S1.pdf]

Table S1. Nucleotide sequences of the primers used in this study

Plasmid	Primer sequence (5′-3′)
pProEx-Hta::CgeA WT	F: GGCCATGGGGATGAGCTCGGAAAACGC<br>R: GGCCTCGAGTTAGCTAAAAAAGGTAACGCTTTC
pProEx-Hta::CgeA A42C	F: CACATTAAAGATATGCTGGAAGCGTGCATTGATCAGAAAATTGATGAAAG<br>R: CTTTCATCAATTTTCTGATCAATGCACGCTTCCAGCATATCTTTAATGTG
pProEx-Hta::CgeA D97C	F: CCCGGTGGTTTTTGTGGCGTTTTGTCAGGTGCATGG<br>R: CCATGCACCTGACAAAACGCCACAAAAACCACCGGG
