# Supplementary material for: Cryo-EM structure of the glycosylated protein CgeA in the crust of Bacillus subtilis endospores
Source: J Microbiol. 2025 Oct 31;63(10):e2504013. doi: 10.71150/jm.2504013 (PMC13577073; doi:10.71150/jm.2504013)
Supplement: Supplementary file 8 [file jm-2504013-Supplementary-Table-S2.pdf]

Table S2. Cryo-electron microscopy data collection and processing statistics

	**Wild-type CgeA**	**CgeA (A42C/D97C)**
**Data collection and processing**		
Magnification	92000	105000
Voltage (kV)	200	300
Frame exposure time (s)	14.62	5.45
Exposure fractionations (n)	50	51
Electron exposure (e^-/Å ^{2})	49.97	63.6
Defocus range (μm)	-2.0 to –1.0	-1.7 to -0.8
Pixel size (Å)	1.1	0.828
Symmetry imposed	C6	C6
Box size (pixel)	150	200
Micrographs collected (n)	1952	10593
Segments extracted (n)	3559545	436793
Segments after Class 2D (n)	661869	88923
Map resolution (Å)	6.8	3.05
FSC threshold	0.143	0.143
**Model building**		
Initial model		AlphaFold 3 model
**Model composition**		
Chains		6
Non-hydrogen atoms		3294
Protein residues		438
**R.M.S deviations**		
Bond lengths (Å)		0.004
Bond angels (°)		0.666
**Validation**		
MolProbity score		1.87
Clash score		10.20
Rotamer outliers (%)		0
Ramachandran plot		
Outliers (%)		0
Allowed (%)		3.44
Favored (%)		96.56
**PDB accession code**		9L9D
**EMDB accession code**		EMD-62902
